# External Application of Traditional Chinese Medicine for Venous Ulcers: A Systematic Review and Meta-Analysis

**DOI:** 10.1155/2015/831474

**Published:** 2015-09-07

**Authors:** Xin Li, Qing-qing Xiao, Kan Ze, Su Li, Yi-fei Wang, Min Zhou, Qin-tong Yang, Fu-lun Li, Bin Li

**Affiliations:** ^1^Department of Dermatology, Yueyang Hospital of Integrated Traditional Chinese and Western Medicine, Shanghai University of Traditional Chinese Medicine, Shanghai 200437, China; ^2^Department of Vessel, Shanghai TCM-Integrated Hospital, Shanghai University of Traditional Chinese Medicine, Shanghai 200082, China

## Abstract

*Objective*. To evaluate the effectiveness of external application of traditional Chinese medicine (EA-TCM) on venous ulcers. *Methods*. Seven databases were searched until April 2015 for randomized controlled trials (RCTs) of EA-TCM for venous ulcers. Risk of bias was assessed using Cochrane Handbook guidelines. Study outcomes were presented as risk ratios (RRs) for dichotomous data or mean differences (MDs) for continuous data. *Results*. Sixteen of 193 potentially relevant trials met the inclusion criteria; however, their methodological qualities were low. Comparison of the same intervention strategies revealed significant differences in total effectiveness rates between EA-TCM and conventional therapy groups (RR = 1.22, 95% confidence interval [CI] = 1.16–1.29, and *P* < 0.00001). Compared to conventional therapy, EA-TCM combined with conventional therapy had a superior total effectiveness rate (RR = 1.11, 95% CI = 1.04–1.19, and *P* = 0.003). There were no significant differences in recurrence rates during followup and final pain measurements between the experimental and those in the control groups (RR = 0.86, 95% CI = 0.31–2.39, and *P* = 0.85; MD −0.75, 95% CI = −2.15–0.65, and *P* = 0.29). *Conclusion*. The evidence that EA-TCM is an effective treatment for venous ulcers is encouraging, but not conclusive due to the low methodological quality of the RCTs. Therefore, more high-quality RCTs with larger sample sizes are required.

## 1. Introduction

The most commonly diagnosed ulcer of the lower extremities, venous leg ulcerations, occurs in approximately 500,000 to 2 million people annually in the United States [1], with a prevalence as high as 4% in populations older than 65 years [2]. The treatment of venous ulcer disease requires significant resources and costs: in the United States, the overall cost is approximately 3 billion dollars per year [3]. The two main objectives of venous ulcer treatment are to heal the ulcer and to avoid ulcer recurrence [4]. Compression and debridement are the standard first-line clinical treatments. Second-line treatments, which involve a range of interventions, are considered when first-line treatments fail. However, until recently, there have not been widely accepted second-line treatment standards.

In addition to surgical treatments for venous ulcers, including more invasive open surgical procedures (i.e., venous ligation and stripping [5]), less invasive open surgical procedures (i.e., ambulatory conservative hemodynamic correction of venous insufficiency (CHIVA) [6] and ablative superficial venous surgery [7]), and less invasive endovenous surgical procedures (i.e., radiofrequency ablation [8] and endovenous laser [5]), therapies collectively known as traditional Chinese medicine (TCM) show a gradual and typically curative effect. In China, TCM has been used to treat human diseases for more than 2000 years. In the history of TCM, physicians have accumulated a tremendous amount of knowledge and experience in treating venous ulcers. As an integral part of TCM, external application of traditional Chinese medicine (EA-TCM) has been perceived as less expensive, safer, and more effective [9–11] than conventional therapies. There are numerous clinical trials regarding the use of EA-TCM for treatment of venous ulcers, with positive results; however, to our knowledge, the potential benefits of EA-TCM for patients with venous ulcers, to justify either their recommendation or their clinical role, have not been evaluated. In addition, a large number of studies could potentially be missed if literature searches are restricted to English-only sources [12]. Therefore, we conducted a systematic review to assess the effect of EA-TCM on venous ulcers.

## 2. Materials and Methods

### 2.1. Data Sources and Searches

To identify relevant randomized clinical trials (RCTs), two reviewers (X. Li and Q. Xiao) systematically searched the Medical Literature Analysis and Retrieval System Online (MEDLINE), Excerpta Medica data BASE (EMBASE), Cochrane Central Register, China National Knowledge Infrastructure database, Chinese Scientific Journals Full Text Database, Wanfang Data Knowledge Service Platform, and the Chinese Biomedical Literature Service System, using the search terms “venous ulcers,” “venous leg ulcer,” “TCM,” “traditional Chinese herb,” “herbal medicine,” “ointment,” and “randomized controlled trial.” In this study, we included papers dating from the earliest citation in the databases until April 2015. The references of all selected publications and reviews were manually searched for further relevant articles. We did not limit publication languages and types, including conference proceedings, abstract-only articles, and theses, as long as they met our inclusion criteria.

### 2.2. Study Selection

#### 2.2.1. Studies

RCTs were included. Quasi-RCTs, non-RCTs, or randomized trials with false randomization methods were excluded.

#### 2.2.2. Participants

Patients diagnosed with venous ulcers based on any set of explicit criteria were included; other ulcers, such as pressure ulcers, were excluded. There were no set limitations on participant age, gender, or nationality.

#### 2.2.3. Interventions

The focused experimental groups received either EA-TCM or EA-TCM combined with conventional therapy. We did not set limitations on dosages, formulations, routes of administration of the traditional Chinese herbs, or types of conventional therapy used.

Our comparison of TCM and conventional therapy included surgical treatment, endovenous surgical procedures, compression therapy, and topical and pharmacological treatment.

#### 2.2.4. Control Group Treatments

Control groups were defined as patients who received any type of conventional therapy for venous ulcers, without TCM treatments.

#### 2.2.5. Outcome Measures

The primary outcomes considered in this study were the total effectiveness rates for the duration of treatment, defined as the rate of change in ulcer size, absolute change in wound size, and number of wounds completely healed. We also evaluated recurrence rates, defined as the detection of new venous ulcers by clinical evaluation after followup. The secondary outcomes included quality of life, pain, and any adverse effects from the interventions.

Trials were excluded if any of the following factors were identified: (1) insufficient information concerning evaluation rates; (2) lack of EA-TCM treatment; (3) mixed interventions in the experimental group (e.g., EA-TCM combined with internal TCM); (4) animal trials.

### 2.3. Data Extraction

Two reviewers (K. Ze and S. Li) extracted data independently using a predefined data extraction form. Disagreements were resolved by discussion or consensus with a third reviewer (B. Li). The data extracted included the first author; study characteristics (i.e., year, duration, setting, and design); participant characteristics (i.e., mean age, sample size, and systemic therapy); external application of the experimental and control group treatments; measured outcomes. For studies with insufficient information, the reviewers contacted the primary authors, when possible, to acquire and verify the data.

### 2.4. Risk of Bias Assessment

The risk of bias in each study was assessed by two independent authors (X. Li and M. Zhou) using the Cochrane Risk of Bias tool [13]; disagreements were resolved either by consensus or by a third reviewer (B. Li).

### 2.5. Data Synthesis and Analyses

For meta-analysis, the total effectiveness rates of dichotomous data were pooled using risk ratios (RRs). All statistical analyses were performed using Review Manager 5.2.1 software (Cochrane Community, London, United Kingdom).

We compared the final results to assess the differences between experimental and control groups. Cochrane's *χ* ^2^ and *I* ^2^ tests were used to assess the degree of heterogeneity between studies. There was considerable heterogeneity for *P* values less than 0.10, or *I* ^2^ value above 50%, in the *χ* ^2^ and *I* ^2^ tests, respectively [13]. In this case, a random-effects model was used in order to compute the global RR and MD. Otherwise, with *P* values greater than 0.10 or *I* ^2^ less than 50%, the between-study heterogeneity was not substantial, and the fixed-effect models were suitable. Clinical heterogeneity was assessed by reviewing the differences in the distribution of participants' characteristics among trials (i.e., age, gender, and duration of disorder and associated diseases).

## 3. Results

### 3.1. Study Selection

**Figure 1:**
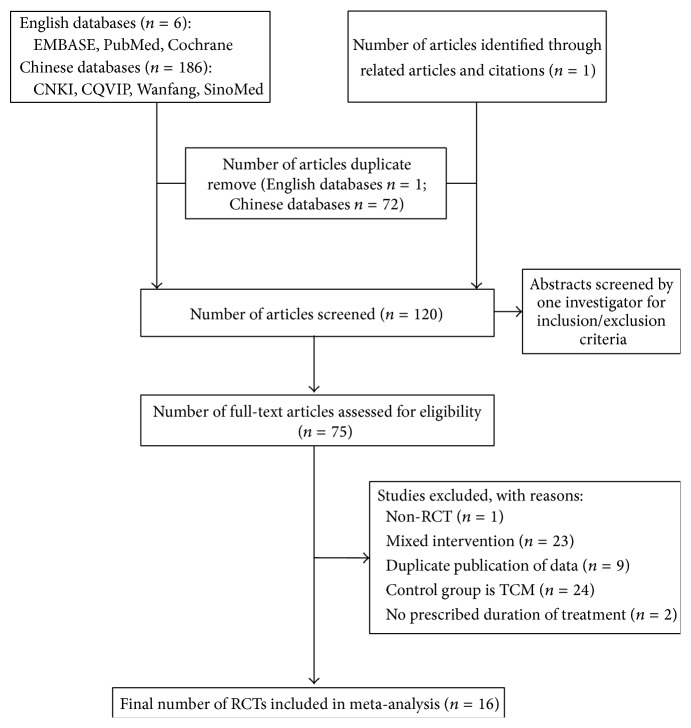
Summary of the literature identification and selection process. CNKI indicates the Chinese National Knowledge Infrastructure database; CQVIP, the Chinese Scientific Journals Full Text database; SinoMed, the Chinese Biomedical Literature Service System; TCM, traditional Chinese medicine; RCT, randomized clinical trials.

From a total of 193 titles, the full text of 75 potentially relevant studies was reviewed to confirm their eligibility. Among these 75 studies, 59 were excluded, including one non-RCT study, 23 with treatments that mixed interventions, nine with duplicate publication of data, 24 that compared treatment intervention with TCM, and two with no prescribed duration of treatment. Finally, 16 trials met the inclusion criteria (Figure 1).

### 3.2. Study Characteristics

All of the 16 trials included in this study were published in Chinese. A total of 1269 participants were included in these trials, with 660 and 609 in the experimental and control groups, respectively. The sample sizes of these trials ranged from 51 to 164. Six trials reported on the adverse events in the experimental group [11, 14–18], while three performed patient followup [11, 14, 19] (Table 1).

The components and suppliers of the traditional Chinese herbs used in each trial varied. The most common form of EA-TCM, used in nine trials, was ointment, including SheXiangZhenZhu [20], KuiYangPing [9], ShengJi [19], moist exposed burn [15], ShengJiYuHong [11, 21], HongYou [18, 22], and FuFangSanHuang ointments [23]. Other forms of EA-TCM used in clinical trials were powders in three trials [10, 14, 16], Chinese-herb external washing in three trials [17, 22, 24], paste in one trial [9], and oil in one trial [25] (Table 2).

### 3.3. Risk of Bias Assessment

**Figure 2:**
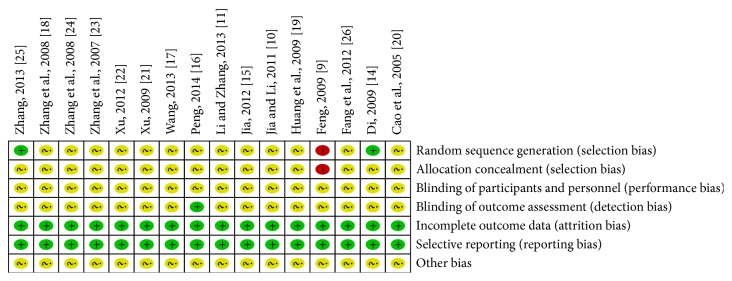
Risk of bias graph.

The methodological quality of all included trials was poor (Figure 2). Although all these trials reported randomization, only three adequately described the randomization method: two with a random number table [14, 25] and one using clinic record numbers [9]. Moreover, none of the studies reported information such as allocation concealment or blinding of participants and study personnel; only one reported the details of the blinding of outcome assessment [16]. All of the relevant trials adequately addressed incomplete outcome data and selective reporting. We found no other biases in these trials; however, considering their poor methodological quality, we determined that an unclear risk of bias should be given to all the included trials.

### 3.4. Primary Outcomes

#### 3.4.1. Total Effectiveness Rates of EA-TCM versus Conventional Therapy Based on the Same Intervention Strategies

**Figure 3:**
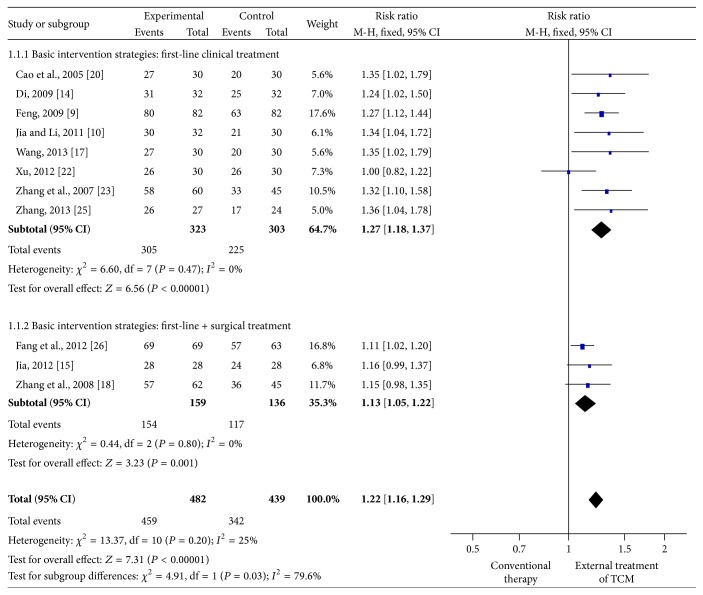
Meta-analysis of the total effectiveness rate of external application of traditional Chinese medicine (EA-TCM) versus conventional therapy based on the same intervention strategies. CI indicates confidence interval.

The 11 RCTs contained 761 patients; the experimental and control groups received EA-TCM and conventional therapy, respectively. All subjects from the two groups received basic intervention strategies, including compression and debridement as first-line clinical treatment and surgical interventions (venous ligation and stripping [15, 26] and endovenous laser [18]) as second-line treatment. Pooling of the results from these trials showed a significant difference in the total effectiveness rate between the EA-TCM and conventional therapy groups (RR = 1.22, 95% confidence interval [CI] = 1.16–1.29, and *P* < 0.00001) using the fixed-effects model. There were also significant differences in each subgroup (basic intervention strategies: first-line clinical treatment RR = 1.27, 95% CI = 1.18–1.37; basic intervention strategies: first-line and surgical treatment RR = 1.13, 95% CI = 1.05–1.22) (Figure 3).

#### 3.4.2. Total Effectiveness Rates of EA-TCM Combined with Conventional Therapy versus Conventional Therapy Alone

**Figure 4:**
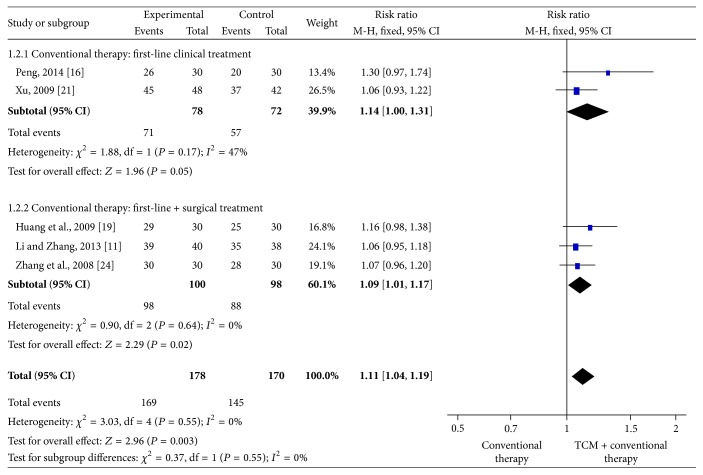
Meta-analysis of the total effectiveness rate of combined external application of traditional Chinese medicine (EA-TCM) and conventional therapy versus conventional therapy alone. CI indicates confidence interval.

Five studies with 314 subjects reported that the experimental groups received EA-TCM combined with conventional therapy and that the control groups received conventional therapy only. Results of meta-analysis using the fixed-effects model indicated a significantly higher total effectiveness rate for EA-TCM combined with conventional therapy compared to that of the control groups (RR = 1.11, 95% CI = 1.04–1.19, and *P* = 0.003). Significant differences were found between subgroups of conventional therapy with first-line clinical treatment (RR = 1.14, 95% CI = 1.00–1.31) and conventional therapy with first-line clinical treatment combined with surgical interventions (RR = 1.09, 95% CI = 1.01–1.17) (Figure 4).

#### 3.4.3. Recurrence Rate Sat Followup

**Figure 5:**
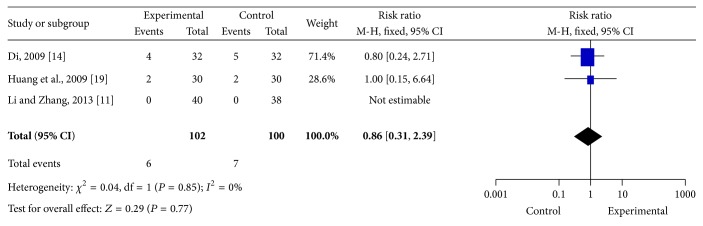
Meta-analysis of recurrence rates during followup. CI indicates confidence interval.

Three studies reported recurrence rates at followup. However, the results of meta-analysis using the fixed-effects model indicated no significant effects in the experimental groups compared to the control groups (RR = 0.86, 95% CI = 0.31–2.39, and *P* = 0.85) (Figure 5).

### 3.5. Secondary Outcomes

**Figure 6:**
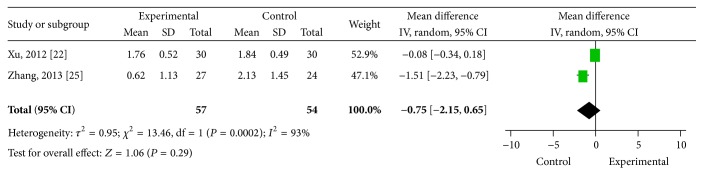
Meta-analysis of final pain measurements. CI indicates confidence interval.

Only two studies reported final pain measurements. With random-effects modelling, the pooled data for the two studies showed a difference between the experimental and control groups in the final pain measurements (MD −0.75, 95% CI = −2.15–0.65, and *P* = 0.29) (Figure 6).

### 3.6. Adverse Events

Six studies reported adverse events in the experimental groups. No significant adverse reactions were noted in three studies [15–17]. Two trials reported two patients with contact dermatitis that resolved with appropriate treatment [14, 18]. Three patients suffered from ecchymosis caused by surgical treatment [11].

### 3.7. Assessment of Publication Bias

In this review, the use of funnel plots was limited due to the small number of studies evaluated.

## 4. Discussion

### 4.1. Summary of Evidence

This review systematically assessed mainly Chinese-sourced RCT studies related to the effects of EA-TCM as a complementary therapy; a total of 16 RCTs were identified for systematic review and meta-analysis. The trials included in this study assessed the efficacy of several types of external application on various medical conditions. A total of 660 patients in treatment groups and 609 in control groups were evaluated, and the duration of RCTs ranged from 6.4 months to 10.5 years. All of these RCTs were conducted in mainland China. Despite the fact that most of the trials had small sample sizes and poor methodological quality, analysis of the pooled data showed a consistently superior effect of EA-TCM or EA-TCM combined with conventional therapy in terms of total effectiveness, when compared to the control groups. There were fewer adverse effects, and none were severe; only two trials mentioned any adverse effects of EA-TCM, in which two patients each presented with slight rashes [14, 18]. No patients dropped out of their trials due to adverse effects, suggesting that EA-TCM is safe for clinical use.

### 4.2. Limitations of This Review

As with all such studies, we acknowledge several limitations. Specifically, the distorting effects of publication and location bias on systematic reviews and meta-analyses have been well documented [27]. Although we are confident that our search strategy located all relevant studies, there remains a certain degree of uncertainty. The quality scores of the included RCTs were generally poor. Although all of the included studies had a randomization design, only three described the details of the randomization [9, 14, 25]. Furthermore, information on allocation concealment or participant and personnel blinding was missing, and only one study reported any details of the blinding of outcome assessments [16].

While Cochrane's *χ* ^2^ and *I* ^2^ tests revealed no statistical heterogeneity in the total effectiveness rate among these studies, an unpredictable clinical heterogeneity was present nonetheless. For example, basic intervention strategies and conventional therapies, components of TCM, wound-cleaning methods, and care approaches were different in each RCT.

### 4.3. Possible Rationales for EA-TCM for Treatment of Venous Ulcers

As a type of chronic skin ulcer, the pathogenesis of venous ulcers is theoretically caused by “Re (heat) evil,” “Yu (qi-stagnancy, blood-stasis),” and “Xu (qi blood and yin yang deficiency)” according to stage. Correspondingly, the treatment principles for the three stages of venous ulcer include clearing away heat and dampness, promoting blood circulation to dissipate blood stasis, and providing supplements for deficiencies [28]. Although the components of EA-TCM used in each trial included in our meta-analysis varied, the treatment principles were consistent: SheXiangZhenZhu ointment [20], QiXing powder [14], ShengJi ointment [19], moist exposed burn ointment [15], and Chinese herbs for external washing [17] all clear away heat and dampness and promote blood circulation to dissipate blood stasis. For example, the main effect of HuangDou paste [26] is to clear away heat and address deficiencies; other treatments [9, 11, 16, 18, 21–23] also offer combined effects for the three treatment stages.

As an important complementary therapy, the use of EA-TCM combined with conventional therapy could offer an effective treatment method for venous ulcers. Therefore, a substantial amount of research has investigated the chemical constituents of EA-TCMs. Moist exposed burn ointment, the most representative EA-TCM, has pharmacological effects that include prevention of dermal water loss, as well as anti-inflammatory, antibacterial, and analgesic properties [29–31]. ShengJiYuHong ointment has been shown to inhibit inflammatory responses by acting on the inflammatory mediators and cells and to improve wound healing by promoting fibroblast proliferation and tissue granulation [32–34]. Wang et al. found that HongYou ointment offers a protective effect on the production and secretion of the extracellular matrix and also promotes fibroblast and endothelial cell proliferation [35].

## 5. Conclusions

While the evidence that EA-TCM may be an effective treatment for venous ulcers is encouraging, it is not conclusive due to the low methodological quality of the RCTs. Therefore, more high-quality RCTs, with low risk of bias and adequate sample sizes, are required to demonstrate its true effects.

## Figures and Tables

**Table 1:** Included RCTs.

Study	Location	Sample size E/C	Age E/C	Duration E/C (months)	Duration of treatment (weeks)	Main outcomes	Evaluation method	Outcome odds [95% CI]; final pain measurement (95%)	ADs of experimental group	FUP (months)
Cao et al., 2005 [20]	China	30/30	63.2/62.5	12.5/12.0	4	TER, CR, IR	Verification of the area	4.50 [1.09–18.50]	NR	NR

Di 2009 [14]	China	32/32	57.75/56.59	44.64/44.16	4	TER, CR, IR	Verification of the area	8.68 [1.00–75.30]	Two patients: contact dermatitis	1

Fang et al., 2012 [26]	China	69/63	59.5/60.5	NR	4	TER, CR, IR	Verification of the area	15.71 [0.87–284.88]	NR	NR

Feng, 2009 [9]	China	82/82	NR	NR	8	TER, CR, IR	Verification of the area	12.06 [2.71–53.74]	NR	NR

Huang et al., 2009 [19]	China	30/30	62	NR	4	TER, CR, IR	Verification of the area; subjective evaluation of a researcher	5.80 [0.63–53.01]	NR	24

Jia and Li, 2011 [10]	China	32/30	55.7/56.6	17.8/17.9	4	TER, CR, IR	Verification of the area	6.43 [1.26–32.83]	NR	NR

Jia, 2012 [15]	China	28/28	53.2	NR	3	TER, CR, IR	Verification of the area; subjective evaluation of a researcher	10.47 [0.54–204.32]	None	NR

Li and Zhang, 2013 [11]	China	40/38	48.6/46.2	10.8/13.2	4	TER, CR, IR	Verification of the area	3.34 [0.33–33.63]	Three patients: ecchymosis	6

Peng, 2014 [16]	China	30/30	65.9/66.4	81.6/82.44	4	TER, CR, IR, VRT	Verification of the area; subjective evaluation of a researcher; PPG	3.25 [0.89–11.90]	None	NR

Wang, 2013 [17]	China	30/30	63.23/63	126/120	4	TER, CR, IR, VRT, VP	Verification of the area; subjective evaluation of a researcher; PPG	4.50 [1.09–18.50]	None	NR

Xu, 2009 [21]	China	48/42	58/57.8	9.5/8.4	2	TER, CR, IR	Verification of the area	2.03 [0.45–9.05]	NR	NR

Xu, 2012 [22]	China	30/30	52.5	NR	4	TER, CR, IR, pain	Verification of the area	1.00 [0.23–4.43]; E 0.62 (1.13); C 2.13 (1.45)	NR	NR

Zhang et al., 2007 [23]	China	60/45	66.7/65.9	11.3/14.9	4	TER, CR, IR	Verification of the area; subjective evaluation of a researcher; photography	10.55 [2.22–50.02]	NR	NR

Zhang et al., 2008 [24]	China	30/30	NR	NR	4	TER, CR, IR	Verification of the area; subjective evaluation of a researcher	5.35 [0.25–116.31]	NR	NR

Zhang et al., 2008 [18]	China	62/45	54.4/52.8	NR	6	TER, CR, IR	Verification of the area; subjective evaluation of a researcher	2.85 [0.88–9.18]	Two patients: contact dermatitis	NR

Zhang, 2013 [25]	China	27/24	59.78/60.05	6.44/7.24	4	TER, CR, IR, pain	Verification of the area; subjective evaluation of a researcher	10.71 [1.21–94.96]; E 1.76 (0.52); C 1.84 (0.49)	NR	NR

RCTs, randomized controlled trials; E, experimental group; C, control group; ADs, adverse events; FUP, follow-up period; NR, no report; TER, total effective rate; CR, curative ratio; IR, inefficiency rate; VRT, venous refill time; PPG, photoplethysmography; VP, venous pressure.

**Table 2:** Treatments used in the included studies.

Study	Conventional therapy of experimental group/control group				Topical treatment of control group	External application of experimental group	Suppliers of the externally applied TCM
	Surgical treatment	Endovenous surgical procedures	Compression therapy	Pharmacological treatment			
Cao et al., 2005 [20]	NA	NA	NR	Antibiotics and nutritional support	Vaseline ointment	SheXiangZhenZhu ointment	The 306th Hospital of Chinese People's Liberation Army

Di, 2009 [14]	NA	NA	Compression bandages or hosiery	NR	Gentamicin	QiXing powder	Chengdu University of TCM

Fang et al., 2012 [26]	VLS	NA	Compression bandages or hosiery	Antibiotics if necessary	Alginate dressing	HuangDou paste	Hospital of Laiwu Iron and Steel Group Limited Company (Chinese Patent Number: ZL 200710115133.0)

Feng, 2009 [9]	NA	NA	NR	NR	Erythromycin ointment	KuiYangPing ointment	Liaoning University of TCM

Huang et al., 2009 [19]	NA	SEPS	NR	NR	NR	ShengJi ointment	The Second Affiliated Hospital of Heilongjiang University of TCM

Jia and Li, 2011 [10]	NA	NA	Compression bandages	NR	0.1% ethacridine lactate	ShengJiYuYang powder	TCM Hospital of Hebei Province

Jia, 2012 [15]	VLS	NA	NR	Antibiotics and nutritional support	Vaseline ointment + gentamicin	Moist exposed burn ointment (MEBO)	Shantou Meibao Pharmaceutical Co., Ltd.

Li and Zhang, 2013 [11]	NA	EVLT	Compression bandages	Antibiotics and aspirin	NR	ShengJiYuHong ointment	TCM Hospital of HanDan city

Peng, 2014 [16]	NA	NA	Compression bandages	Danhong injection; elastic bandage	0.1% ethacridine lactate	0.1% ethacridine lactate + Chinese herbal powder	TCM Hospital of Hebei Province

Wang, 2013 [17]	NA	NA	IPC + Compression bandages	Salvia miltiorrhiza and ligustrazine injection	ethacridine lactate	Chinese herbs external washing	TCM Hospital of Hebei Province

Xu, 2009 [21]	NA	NA	NR	Antibiotics	NR	ShengJiYuHong ointment	TCM Hospital of Jiangsu Province

Xu, 2012 [22]	NA	NA	NR	NR	Ethacridine lactate	Chinese herbs external washing + HongYou ointment	Shuguang Hospital, Shanghai University of TCM

Zhang et al., 2007 [23]	NA	NA	Compression hosiery	Antibiotics if necessary and nutritional support	Vaseline ointment	FuFangSanHuang ointment	TCM Hospital of Shijiazhuang City

Zhang et al., 2008 [24]	VLS	NA	Compression hosiery	NR	NR	Chinese herbs external washing	People's Hospital of Yunnan Chuxiong

Zhang et al., 2008 [18]	NA	EVLT	Compression bandages	NR	Sensitive antibiotics	HongYou ointment	Shuguang Hospital, Shanghai University of TCM

Zhang, 2013 [25]	NA	NA	NR	NR	Metronidazole	KuiYang oil	Dongzhimen Hospital, Beijing University of Chinese Medicine

NA, not available; NR, no report; TCM, traditional Chinese medicine; VLS, venous ligation and stripping; SEPS, subfascial endoscopic perforator vein surgery; EVLT, endovenous laser treatment; IPC, intermittent pneumatic compression.
